# Digital Health and Artificial Intelligence Continuing Professional Development Needs Among Rehabilitation Professionals in Jordan: A National Mixed-Methods Study

**DOI:** 10.3390/healthcare14203403

**Published:** 2026-10-12

**Authors:** Fadi Almasalha, Mohammad Z. Darabseh, Musab Abu Beidar, Bodor Bin Sheeha, Shruoq Faruoq Husain Jebreel, Arar Al Tawil, Sami Elmahgoub, Wesam A. Debes, Mohammad Mabrouk, Badrieh Al Abbad, Aseel Aburub

**Affiliations:** 1 Department of Computer Science, Faculty of Information Technology, Applied Science Private University, Amman 11937, Jordan; 2 Department of Physiotherapy, School of Rehabilitation Sciences, University of Jordan, Amman 11942, Jordan; 3 Department of Physiotherapy, Faculty of Allied Medical Sciences, Applied Science Private University, Amman 11937, Jordan; 4 Department of Rehabilitation Sciences, College of Health and Rehabilitation Sciences, Princess Nourah bint Abdulrahman University, Riyadh 11671, Saudi Arabia; bhbinsheeha@pnu.edu.sa; 5 Department of Continuous Education, High Health Council, Amman 11941, Jordan; 6 Computer Sciences Department, Faculty of Information Technology, Applied Science Private University, Amman 11937, Jordan; 7 School of Allied Health Professions, Keele University, Keele ST5 5BG, Staffordshire, UK

**Keywords:** continuing professional development (CPD), digital health, artificial intelligence, rehabilitation workforce, rehabilitation professionals, telerehabilitation, digital competencies, mixed-methods research, Jordan, low- and middle-income countries

## Abstract

Background: Digital health and artificial intelligence (AI) are rapidly transforming rehabilitation practice, creating an urgent need for rehabilitation professionals to develop new competencies through continuing professional development (CPD). Despite the growing importance of digital rehabilitation, evidence regarding digital health and AI-related CPD needs among rehabilitation professionals in low- and middle-income countries remains limited. This study aimed to identify the digital health and AI-related CPD needs of rehabilitation professionals in Jordan. Methods: A national convergent parallel mixed-methods study was conducted between January and May 2026. Licensed physiotherapists, occupational therapists, speech-language therapists, and prosthetists/orthotists completed a structured online questionnaire assessing CPD participation, digital health competencies, AI-related knowledge, perceived barriers, and future educational priorities. Semi-structured interviews were conducted with a purposive sample of rehabilitation professionals and key stakeholders to further explore experiences and educational needs. Quantitative data were analyzed using descriptive and inferential statistics, while qualitative data underwent reflexive thematic analysis. Findings from both components were integrated during interpretation. Results: A total of 462 rehabilitation professionals completed the survey, and 20 participated in qualitative interviews. Artificial intelligence (mean = 4.71 ± 0.52), telerehabilitation (4.64 ± 0.61), clinical decision support systems (4.59 ± 0.65), and virtual reality rehabilitation (4.55 ± 0.69) emerged as the highest-priority CPD topics. Among the 462 participants, 169 (36.6%) reported previous participation in digital-health-related CPD, while 82 (17.7%) reported receiving formal education in artificial intelligence. The principal barriers to CPD participation were lack of time (84.4%), financial cost (79.2%), limited AI-focused educational opportunities (76.8%), and inadequate employer support (63.0%). Previous digital health training, years of professional experience, and employer support independently predicted higher self-reported digital health competency (adjusted R^2^ = 0.39; *p* < 0.05). Qualitative findings demonstrated that rehabilitation professionals recognized digital transformation as inevitable but reported insufficient practical training, organisational support, and nationally coordinated educational opportunities. Conclusions: Jordanian rehabilitation professionals demonstrated high motivation to develop competencies in digital health and AI but reported substantial educational and organisational barriers. National competency frameworks, structured CPD programmes, and integration of digital health into rehabilitation curricula are needed to prepare the rehabilitation workforce for technology-enabled healthcare. The findings may inform workforce development strategies in Jordan and other low- and middle-income countries undergoing digital transformation.

## 1. Introduction

Rehabilitation services help people recover function, maximize independence, and improve quality of life following injury, illness, or disability [1,2]. Globally, the need for rehabilitation is increasing, with an estimated 2.4 billion people currently living with health conditions that could benefit from rehabilitation, while in many low- and middle-income countries (LMICs), more than 50% of those requiring rehabilitation do not receive these services [1,2]. Meeting this growing demand is a core component of Universal Health Coverage (UHC), and the rehabilitation workforce, including physiotherapists, occupational therapists, speech-language therapists, audiologists, prosthetists, and orthotists, is essential to achieving this goal [1,3]. These professionals must continually update their knowledge and competencies to keep pace with evolving evidence, technologies, and models of care through continuing professional development (CPD) [4]. In Jordan, CPD has recently become mandatory for health professional licensure, reflecting the country’s commitment to improving healthcare quality [5]. Madi and Hamzeh [5] found that Jordanian physiotherapists primarily participate in CPD through workshops, journal reading, and workplace lectures, motivated mainly by improving professional knowledge and patient care. However, they also identified major barriers, including financial cost, travel requirements, and limited time, while similar evidence remains unavailable for other rehabilitation professions in Jordan [5].

At the same time, digital health innovations are transforming rehabilitation practice, service delivery, and professional education worldwide [1]. The COVID-19 pandemic accelerated the implementation of telehealth and virtual rehabilitation (VRehab), and growing evidence indicates that well-designed home-based digital rehabilitation can achieve clinical outcomes comparable to conventional face-to-face interventions for many patient populations [1,6]. These technologies generate extensive clinical data, including motion sensor outputs, wearable device data, and video recordings, that are increasingly analyzed using artificial intelligence (AI) to evaluate movement quality, predict functional outcomes, personalize rehabilitation programs, and support clinical decision-making [6,7]. Reviews of health professions education have also demonstrated rapid expansion in digital learning modalities, including online learning platforms, simulation, and virtual reality, although most available evidence originates from high-income countries, highlighting an important evidence gap for LMICs [1].

Evidence from Jordan similarly points to workforce challenges. A recent qualitative study of e-health implementation in Jordan identified digital skills and training gaps among healthcare professionals as a key barrier to adoption, and emphasised structured education and training as a prerequisite for successful implementation [8]. Likewise, reviews of telerehabilitation implementation in the Middle East indicate that rehabilitation professionals generally recognize the value of virtual care but report limited practical experience and identify training, technological infrastructure, and supportive policies as prerequisites for successful implementation [9]. Collectively, these findings underscore the urgent need for Jordanian rehabilitation professionals to develop competencies in emerging digital health technologies.

In particular, important CPD priorities may include AI-assisted rehabilitation, virtual reality and immersive rehabilitation, telerehabilitation, wearable sensors and digital outcome measures, and clinical decision-support systems, all of which are increasingly incorporated into modern rehabilitation practice [1,7]. Despite this technological transformation, no national study has comprehensively examined the digital and AI-related CPD needs of Jordanian rehabilitation professionals. Therefore, this mixed-methods national study aims to identify the digital health and AI-related CPD requirements of physiotherapists, occupational therapists, speech-language therapists, and prosthetists/orthotists in Jordan. The findings will inform future CPD programs, workforce development strategies, and national policies designed to prepare the rehabilitation workforce for technology-enabled rehabilitation practice.

## 2. Materials and Methods

### 2.1. Study Design

This study employed a convergent parallel mixed-methods design to comprehensively assess the continuing professional development (CPD) needs related to digital health and artificial intelligence (AI) among rehabilitation professionals in Jordan. In a convergent mixed-methods design, quantitative and qualitative data are collected during the same phase of the study, analyzed independently, and subsequently integrated to provide a comprehensive understanding of the research problem [10]. This design was selected because quantitative methods enabled the identification of the prevalence and distribution of CPD needs across rehabilitation professions, whereas qualitative methods provided an in-depth understanding of participants’ experiences, perceptions, and contextual factors influencing self-reported digital health competency development [11].

The study was guided by the Good Reporting of A Mixed Methods Study (GRAMMS) framework to ensure methodological rigor and transparency in the design, implementation, analysis, and reporting of mixed-methods research [12]. The quantitative component followed the Strengthening the Reporting of Observational Studies in Epidemiology (STROBE) recommendations for cross-sectional studies [13], while the qualitative component adhered to the Consolidated Criteria for Reporting Qualitative Research (COREQ) checklist [14].

The quantitative component consisted of a national cross-sectional survey designed to identify rehabilitation professionals’ current participation in CPD activities, perceived educational priorities, digital health competencies, AI-related knowledge, and barriers to engaging in technology-focused professional development. The qualitative component comprised semi-structured interviews with rehabilitation professionals and key stakeholders to explore these issues in greater depth, clarify survey findings, and identify contextual factors influencing future workforce development.

### 2.2. Study Setting

The study was conducted between January 2026 and May 2026 across the Hashemite Kingdom of Jordan. Participants were recruited from governmental hospitals, private hospitals, rehabilitation centers, university hospitals, primary healthcare centers, private rehabilitation clinics, academic institutions, and non-governmental organizations providing rehabilitation services.

To achieve broad national geographic coverage, recruitment was undertaken across the North, Central, and South regions of Jordan. Because convenience and snowball sampling were used, this geographic coverage should not be interpreted as evidence of national representativeness. Eligible participants included rehabilitation professionals practicing in urban and rural settings, thereby capturing variations in workplace characteristics, accessibility to CPD opportunities, and digital health infrastructure.

The study targeted the principal rehabilitation professions recognized within the Jordanian healthcare system, including physiotherapists, occupational therapists, speech-language therapists, prosthetists, and orthotists. These professions represent the core rehabilitation workforce identified by the World Health Organization as essential for strengthening rehabilitation services and achieving Universal Health Coverage [1].

### 2.3. Participants and Eligibility Criteria

The study population consisted of licensed rehabilitation professionals currently practicing in Jordan. Four rehabilitation professions were included: physiotherapists, occupational therapists, speech-language therapists, and prosthetists/orthotists. These professions were selected because they represent the principal rehabilitation disciplines identified by the World Health Organization as essential components of rehabilitation service delivery [1].

Participants were eligible if they were registered with the relevant professional body or licensing authority in Jordan, had at least one year of professional clinical experience, were actively practicing in governmental, private, academic, or non-governmental healthcare settings during the study period, and were able to read and understand English sufficiently to complete the quantitative questionnaire. For participation in the qualitative interviews, participants could communicate in either Arabic or English according to their preference. Rehabilitation professionals who were undergraduate students, interns, retired, unemployed, or practicing outside Jordan at the time of data collection were excluded from the study.

For the qualitative component, additional purposive sampling criteria were applied to ensure variation in profession, years of clinical experience, workplace sector, geographical location, previous participation in CPD activities, and experience with digital health technologies. This approach facilitated the inclusion of participants with diverse perspectives and experiences, thereby enhancing the richness and credibility of the qualitative findings [15].

### 2.4. Sampling Strategy

A convergent mixed-methods sampling strategy was employed. The quantitative component used a cross-sectional survey distributed nationally using a combination of convenience and snowball sampling techniques. Recruitment was facilitated through professional associations, healthcare institutions, universities, rehabilitation centers, and professional social media platforms. Invitation messages containing the study information sheet and survey link were disseminated via email, WhatsApp, Facebook and professional networking groups commonly used by rehabilitation professionals in Jordan.

Because no comprehensive national registry containing accessible contact information for all rehabilitation professionals was available, probability sampling was not feasible. Consequently, a non-probability sampling approach was considered the most practical method for achieving broad national representation across professions and healthcare sectors. Similar recruitment strategies have been widely employed in national surveys investigating healthcare professionals’ continuing professional development, digital health competencies, and workforce educational needs [5,16].

For the qualitative component, purposive sampling was used to recruit information-rich participants capable of providing detailed insights into digital health education and professional development needs [17,18].

### 2.5. Sample Size Determination

The required sample size for the quantitative survey was estimated using Cochran’s formula for cross-sectional studies involving categorical data [19]. Assuming a 95% confidence level, a 5% margin of error, and a conservative population proportion of 50%, the minimum required sample size was calculated as 384 participants. The use of a 50% estimated proportion maximizes sample size when the true prevalence of the outcome is unknown and is widely recommended for cross-sectional survey research [19].

To compensate for incomplete responses and missing data, the target recruitment number was increased by approximately 20%, resulting in a minimum recruitment target of approximately 460 participants. This strategy was adopted to preserve statistical power while ensuring adequate representation across the four rehabilitation professions.

The adequacy of the sample size for multivariable analyses was also considered. Recommendations suggest a minimum of 10 to15 observations per predictor variable when performing multiple regression analyses, thereby reducing the risk of model overfitting and unstable parameter estimates [20]. The achieved sample exceeded these recommendations and therefore was considered sufficient for the planned statistical analyses.

No predetermined sample size was established for the qualitative component. Recruitment and analysis proceeded iteratively, with attention to the richness and diversity of participants’ accounts and the extent to which the developing dataset addressed the study aims. By the eighteenth interview, no substantially new patterns relevant to the research questions were being identified, and two additional interviews were conducted to assess the stability and richness of the developing themes. Twenty interviews were therefore completed. Recent methodological evidence suggests that meaning saturation in relatively homogeneous populations is commonly achieved after approximately 16 to 24 interviews, although additional interviews may be required when participant diversity is intentionally sought [18]. Consequently, interviews continued until no new themes emerged across successive interviews.

### 2.6. Questionnaire Development

The questionnaire was developed specifically for this study to assess continuing professional development needs related to digital health and artificial intelligence among rehabilitation professionals. Item generation was informed by a review of the literature addressing digital health competencies, artificial intelligence literacy, digital rehabilitation, continuing professional development, technology acceptance, and barriers to professional education. The questionnaire domains were designed to address six major areas: current CPD participation, barriers to CPD, self-reported digital health competency, AI-related knowledge and critical appraisal, CPD priorities, and preferred CPD delivery methods.

An initial pool of 67 items was generated by the research team. Eight experts reviewed the preliminary questionnaire, including three rehabilitation academics, two clinical rehabilitation specialists, one digital health specialist, one health-professions education specialist, and one biostatistician. Experts evaluated item relevance, clarity, comprehensiveness, and appropriateness using a four-point rating scale. Items with a content validity index below 0.78 were revised or removed. Following expert review, 61 items were retained.

The revised questionnaire was pilot-tested among 30 rehabilitation professionals who were not included in the final study sample. Participants were asked to comment on clarity, comprehensibility, response options, questionnaire length, and completion burden. The pilot required approximately 16 min to complete. Minor modifications were subsequently made to the wording of five items, the sequencing of three items, and the instructions for the Likert-scale response options. No substantive domain was added or removed following pilot testing.

The questionnaire was administered in English. Participants were required to demonstrate sufficient ability to read and understand English to complete the questionnaire. Qualitative interviews were conducted in either Arabic or English according to participant preference, and Arabic interviews were translated into English before analysis where necessary. The final questionnaire is provided in Appendix A.

The psychometric evaluation included assessment of internal consistency using Cronbach’s alpha and examination of the underlying factor structure using exploratory factor analysis. These procedures provide evidence regarding reliability and internal structure but do not constitute complete validation of the instrument. Further validation using independent samples and confirmatory analysis is recommended.

Employer support was assessed using the five-point Likert-scale item ‘My employer provides inadequate support.’ Responses were reverse-coded so that higher scores indicated greater perceived employer support. Previous participation in digital health CPD was coded as 0 = no and 1 = yes. Years of professional experience were coded continuously in years. Categorical variables were dummy-coded using the largest or conceptually appropriate reference category. Regression coefficients are reported with standard errors, standardised coefficients, 95% confidence intervals, and *p*-values.

The AI-related questionnaire section was conceptualised as a multidimensional domain rather than a direct measure of objectively demonstrated AI competency. Items assessed foundational AI knowledge, understanding of terminology, recognition of rehabilitation applications, awareness of limitations, critical evaluation of AI-generated recommendations, ethical and privacy awareness, self-reported digital health competency using AI-assisted rehabilitation tools, and willingness to use AI following appropriate training. The latter item was interpreted as an indicator of technology acceptance/readiness rather than competence. This distinction was maintained when interpreting the quantitative and qualitative findings.

Prompt engineering for healthcare was included as an emerging applied skill because rehabilitation professionals may increasingly interact with generative AI systems through natural-language instructions. However, prompt engineering should not be regarded as a standalone indicator of AI competency. Foundational AI competencies are more broadly concerned with understanding AI limitations, critically evaluating AI-generated outputs, recognising bias and uncertainty, protecting patient data, maintaining human oversight, understanding accountability, and evaluating clinical safety. Accordingly, prompt engineering should be positioned within a broader responsible-AI curriculum rather than treated as an independent measure of competence.

### 2.7. Data Collection Procedures

Data were collected between January 2026 and May 2026 using an anonymous online questionnaire created in Microsoft Forms. The survey link was distributed electronically through professional associations, healthcare institutions, universities, rehabilitation centers, and professional social media platforms. Invitation messages included a participant information sheet describing the study objectives, eligibility criteria, voluntary nature of participation, estimated completion time, and contact details of the principal investigator.

Electronic informed consent was obtained before participants accessed the questionnaire. Participants could complete the survey only once, and no personally identifiable information was collected. Responses were automatically stored on a secure password-protected server accessible only to the research team.

To maximize participation, reminder invitations were disseminated at two-week intervals throughout the data collection period. The questionnaire required approximately 15 to 20 min to complete.

For the qualitative component, eligible participants who indicated their willingness to participate in follow-up interviews were purposively selected to ensure diversity in profession, years of experience, workplace sector, and geographical region. Semi-structured interviews were conducted either face-to-face or through a secure videoconferencing platform according to participant preference. Each interview lasted approximately 30 to 60 min and was conducted by the principal investigator, who had previous qualitative research experience.

An interview guide developed from the literature and preliminary survey findings was used to facilitate discussion. Key topics included participants’ experiences with continuing professional development, perceived digital health competencies, attitudes toward artificial intelligence, barriers to technology adoption, educational priorities, and recommendations for future CPD initiatives.

All interviews were audio-recorded with participant consent and transcribed verbatim. Arabic interviews were translated into English where necessary for analysis. Field notes were maintained throughout data collection to document contextual observations and emerging analytical insights.

### 2.8. Quantitative Data Analysis

Quantitative data were analyzed using IBM SPSS Statistics version 23 (IBM Corp., Armonk, NY, USA). Prior to analysis, data were screened for completeness, missing values, outliers, and normality. Continuous variables were assessed for distributional characteristics before analysis. Normally distributed variables were summarised using mean ± SD, whereas skewed variables were summarised using median and interquartile range (IQR). Age was approximately normally distributed and was therefore reported as mean ± SD (34.8 ± 8.7 years), whereas years of professional experience demonstrated mild positive skewness and was therefore reported as median (IQR) (8 years [4,5,6,7,8,9,10,11,12,13,14]). Internal consistency of the multi-item questionnaire domains was assessed using Cronbach’s alpha. The underlying factor structure was examined using exploratory factor analysis (EFA). Sampling adequacy was assessed using the Kaiser–Meyer–Olkin (KMO) statistic and Bartlett’s test of sphericity. Principal axis factoring with Promax oblique rotation was used because the questionnaire domains were expected to be conceptually related. Factors were retained on the basis of eigenvalues greater than 1.0, inspection of the scree plot, and parallel-analysis considerations. Items with factor loadings ≥0.40 were considered salient, while cross-loadings ≥0.30 were examined for conceptual ambiguity. The final four-factor solution explained 71.4% of the total variance. Detailed factor loadings, cross-loadings, eigenvalues, and item allocation are provided in Supplementary Table S1. Confirmatory factor analysis was not conducted because the present study focused on exploratory development and initial evaluation of the questionnaire. Bivariate analyses were performed to examine differences in CPD needs and self-reported digital health competency scores across participant characteristics. Independent-samples *t*-tests or Mann–Whitney *U* tests were used for comparisons between two groups, whereas one-way analysis of variance (ANOVA) or Kruskal–Wallis tests were used when comparing three or more groups, depending on data distribution. Associations between categorical variables were examined using Pearson’s chi-square test or Fisher’s exact test where appropriate.

Variables demonstrating statistical significance during bivariate analyses or considered clinically relevant based on previous literature were entered into multivariable regression models to identify independent predictors of digital health competency and AI-related CPD needs. Multicollinearity was assessed using variance inflation factors (VIF), and model assumptions were examined before final model interpretation. Statistical significance was established at *p* < 0.05.

### 2.9. Qualitative Data Analysis

Qualitative data were analysed using reflexive thematic analysis following Braun and Clarke [21,22]. The analysis was inductive and interpretive. The researchers first familiarised themselves with the transcripts and maintained reflexive notes documenting initial observations, assumptions, and emerging interpretations. Initial coding was undertaken iteratively by the principal researcher, with regular analytic discussions with members of the research team used to explore alternative interpretations, refine candidate themes, and challenge assumptions. These discussions were intended to enhance reflexivity and interpretive depth rather than to establish inter-rater reliability or consensus coding. Themes were iteratively reviewed against the original transcripts and refined to ensure internal coherence and clear conceptual distinction.

The principal interviewer was a rehabilitation academic with previous qualitative research experience and professional familiarity with rehabilitation education and digital health. The research team remained aware that their professional experiences could influence interpretation of participants’ accounts. Reflexive notes and regular analytic discussions were therefore used to identify and challenge assumptions during the development of themes. NVivo was used to organise transcripts, codes, and supporting quotations.

### 2.10. Trustworthiness of the Qualitative Findings

The trustworthiness of the qualitative findings was established according to the criteria proposed by Lincoln and Guba [23], including credibility, dependability, confirmability, and transferability.

Credibility was enhanced through prolonged engagement with the data, investigator triangulation, and member checking, whereby participants were invited to review summaries of the interview findings for accuracy and completeness. Dependability was strengthened by maintaining a detailed audit trail documenting methodological decisions throughout the study.

Confirmability was supported through researcher reflexivity, maintenance of reflexive notes, and regular analytic discussions within the research team to identify and critically examine assumptions and alternative interpretations. Transferability was enhanced by providing detailed descriptions of participant characteristics, study setting, sampling procedures, and analytical methods to enable readers to determine the applicability of findings to other settings.

### 2.11. Ethical Considerations

Ethical approval was obtained from the Institutional Review Board of the Faculty of Allied Medical Sciences at Applied Science Private University (Approval No. AMS-2025-26, approved on 12 October 2025 before commencement of the study. All procedures were conducted in accordance with the ethical principles outlined in the Declaration of Helsinki.

Participation was entirely voluntary. Electronic informed consent was obtained from all participants before data collection, and participants were informed of their right to withdraw from the study at any time without consequence.

Survey responses were collected anonymously, and interview transcripts were de-identified before analysis. All electronic data were stored on password-protected computers accessible only to the research team. Audio recordings were permanently deleted following transcription and verification, and all study data will be retained for five years after publication in accordance with institutional research governance policies.

## 3. Results

### 3.1. Participant Characteristics

A total of 462 rehabilitation professionals completed the questionnaire. Of the 462 participants, 77 (16.7%) were from the North, 312 (67.5%) from the Central region, and 73 (15.8%) from the South. The predominance of participants from the Central region reflects the concentration of rehabilitation institutions and professional networks in the capital and surrounding areas. The largest professional group was physiotherapists (n = 212, 45.9%), followed by occupational therapists (n = 96, 20.8%), speech-language therapists (n = 84, 18.2%), and prosthetists/orthotists (n = 70, 15.2%). The mean age was 34.8 ± 8.7 years and the median clinical experience was 8 years. Table 1 represents the characteristics of the quantitative study participants.

Self-reported digital health competency differed significantly across professional groups (one-way ANOVA, F = 6.84, *p* < 0.001, η^2^ = 0.043). Physiotherapists reported the highest mean score (3.58 ± 0.61), followed by occupational therapists (3.41 ± 0.65), speech-language therapists (3.29 ± 0.67), and prosthetists/orthotists (3.18 ± 0.70). Post-hoc comparisons indicated significantly higher scores among physiotherapists compared with speech-language therapists and prosthetists/orthotists. These differences should be interpreted cautiously because they may reflect differences in prior exposure to digital technologies, educational opportunities, and workplace context rather than inherent profession-specific capability.

### 3.2. Reliability and Construct Validity

Internal consistency was excellent (overall Cronbach’s α = 0.93). Domain α values were: Digital Health Competency = 0.91, AI Competency = 0.89, CPD Barriers = 0.86, and CPD Needs = 0.94. KMO = 0.92 and Bartlett’s test was significant (*p* < 0.001). EFA identified four factors explaining 71.4% of the variance. Exploratory factor analysis demonstrated excellent sampling adequacy (KMO = 0.92), and Bartlett’s test of sphericity was significant (χ^2^ = 6842.31, df = 595, *p* < 0.001), indicating that the correlation matrix was suitable for factor analysis. Principal axis factoring with Promax rotation produced a four-factor solution. The retained factors had eigenvalues of 8.92, 6.31, 5.17, and 4.86 and accounted for 28.4%, 20.1%, 12.7%, and 10.2% of the variance, respectively, explaining 71.4% of the total variance. Factor loadings ranged from 0.68 to 0.86, and no item demonstrated a problematic cross-loading above 0.30 on more than one factor. The four factors were interpreted as Digital Health Practice, AI Knowledge and Critical Appraisal, CPD Barriers and Organisational Support, and Digital Health CPD Priorities.

### 3.3. CPD Participation and Needs

Workshops (81.6%) and webinars (68.8%) were the most frequently attended CPD activities. The highest-priority future topics were Artificial Intelligence (mean = 4.71 ± 0.52), Telerehabilitation (4.64 ± 0.61), Clinical Decision Support Systems (4.59 ± 0.65), and Virtual Rehabilitation (4.55 ± 0.69).

### 3.4. Barriers

The most frequently reported barriers were lack of time (84.4%), high financial cost (79.2%), limited availability of AI-focused CPD (76.8%), inadequate employer support (63.0%), and travel constraints (58.9%).

### 3.5. Inferential Analysis

Participants with previous digital health training demonstrated significantly higher self-reported digital health competency scores (4.12 ± 0.51 vs. 3.38 ± 0.62; t = 12.84, *p* < 0.001). Physiotherapists reported significantly greater confidence in digital technologies than other professions (F = 5.83, *p* = 0.001). Multiple linear regression showed that previous digital health training (β = 0.41, *p* < 0.001), years of experience (β = 0.19, *p* = 0.008), and employer support (β = 0.24, *p* < 0.001) independently predicted self-reported digital health competency (Adjusted R^2^ = 0.39).

The multivariable model explained 39% of the adjusted variance in self-reported digital health competency, indicating that substantial individual and organisational variability remained unexplained. Potential determinants not fully captured by the current model may include age, gender, workplace setting, quality of internet access, previous exposure to digital technologies, baseline digital literacy, access to digital equipment, and the digital maturity of the employing organisation. Some of these factors were not directly measured and therefore could not be evaluated. The model should consequently be interpreted as an estimate of adjusted cross-sectional associations rather than a comprehensive explanatory model. Table 2 represent the multivariable linear regression for self-reported digital health competency.

### 3.6. Qualitative Findings

Twenty semi-structured interviews were conducted using purposive maximum-variation sampling. The sample included physiotherapists (n = 6), occupational therapists (n = 5), speech-language therapists (n = 4), prosthetists/orthotists (n = 3), and academic/managerial stakeholders (n = 2). Participants represented the North, Central, and South regions and varied in years of experience, workplace sector, and previous exposure to digital health. By the eighteenth interview, no substantially new patterns relevant to the research questions were being identified; two additional interviews were therefore conducted to assess the stability and richness of the developing themes. The qualitative sample was not intended to proportionally reproduce the quantitative sample but was designed to maximise diversity and information richness. (Table 3). Although participants represented different professions and practice settings, there was remarkable consistency regarding the perceived need for digital health and AI-related CPD. Differences between professions primarily reflected variations in previous exposure to digital technologies rather than differences in attitudes toward innovation.

Theme 1. Rehabilitation professionals recognised digital transformation as inevitable but felt inadequately prepared.

Participants consistently described digital transformation as an unavoidable component of future rehabilitation practice. Most believed that digital technologies, including telehealth, wearable devices, virtual reality, and AI-assisted clinical decision-making, would fundamentally change rehabilitation services over the coming decade. Despite recognising these developments, nearly all participants acknowledged feeling underprepared to integrate these technologies into routine clinical practice.

Subtheme 1.1 Awareness exceeded competence.

Participants were generally aware of emerging technologies through conferences, social media, or scientific literature; however, this awareness rarely translated into practical competence.

“We hear about artificial intelligence almost every week, but knowing about it and actually using it with patients are completely different things.”

(Physiotherapist, 9 years)

Several participants explained that they had observed demonstrations of digital rehabilitation technologies but had never received structured training on implementation.

“I have attended presentations on virtual rehabilitation, but I have never had the opportunity to use these systems with real patients.”

(Occupational Therapist, 11 years)

Subtheme 1.2 Fear of being left behind.

Many participants expressed concern that rehabilitation professionals who failed to acquire digital competencies would struggle to remain competitive in future healthcare systems.

“Healthcare is changing rapidly. If we continue practising exactly as we did ten years ago, we will eventually become outdated.”

(Speech-Language Therapist, 15 years)

Theme 2. Current CPD opportunities did not adequately address emerging technologies.

Participants consistently reported that existing CPD programmes focused predominantly on traditional clinical skills, while structured education in digital health and AI remained scarce.

Subtheme 2.1 Traditional CPD dominated available training.

Although workshops and conferences were widely available, they rarely addressed advanced digital technologies.

“Most courses still teach conventional assessment and treatment techniques. Very few discuss artificial intelligence or digital rehabilitation.”

(Physiotherapist, 6 years)

Participants indicated that digital topics were often introduced only superficially without practical application.

Subtheme 2.2 Demand for practical learning experiences.

Rather than traditional lectures, participants overwhelmingly preferred experiential learning approaches.

“Watching someone explain AI is not enough. We need to practise using these systems ourselves.”

(Occupational Therapist, 8 years)

Simulation-based education, virtual reality demonstrations, case discussions, and supervised clinical workshops were repeatedly identified as preferred educational methods.

Theme 3. Organisational and system-level barriers limited self-reported digital health competency development.

Participants identified multiple barriers operating at individual, organisational, and national levels.

Subtheme 3.1 Time and workload.

Heavy clinical workloads were consistently described as the principal obstacle to CPD participation.

“By the time my working day finishes, I simply don’t have the energy to attend another three-hour course.”

(Speech-Language Therapist, 12 years)

Several participants highlighted that digital health training required substantial time commitments beyond routine clinical duties.

Subtheme 3.2 Limited institutional support.

Many participants believed that employers encouraged innovation conceptually but provided insufficient practical support.

“Management expects us to use digital technologies, yet there is no protected learning time or financial support for training.”

(Prosthetist, 14 years)

Participants also described inadequate access to equipment, unreliable internet connectivity, and limited technical support as barriers to implementation.

Theme 4. Artificial intelligence was viewed as a clinical support tool rather than a replacement for professional judgement.

Participants generally expressed positive attitudes toward AI while emphasizing that technology should complement, rather than replace, clinical reasoning.

Subtheme 4.1 AI as an opportunity to improve clinical efficiency.

Participants anticipated that AI could reduce administrative workload, facilitate documentation, monitor patient progress, and support evidence-informed clinical decision-making.

“If AI could analyse patient progress and prepare reports automatically, I would have much more time to focus on treatment.”

(Physiotherapist, 10 years)

Subtheme 4.2 Ethical concerns remained prominent.

Despite enthusiasm for AI, participants consistently discussed ethical issues surrounding privacy, transparency, accountability, and potential bias.

“If an AI system recommends a treatment that harms a patient, who is responsible? These questions need to be answered before widespread implementation.”

(Academic Participant)

Many participants believed that ethics should constitute an essential component of future CPD programmes.

Theme 5. Participants advocated for a national digital rehabilitation CPD strategy.

The final theme reflected participants’ desire for coordinated national leadership rather than isolated educational initiatives.

Subtheme 5.1 Standardised national competencies.

Participants recommended establishing national competency standards for digital rehabilitation practice.

“Every rehabilitation professional in Jordan should graduate with the same minimum digital competencies.”

(Academic Participant)

Subtheme 5.2 Collaboration between universities, professional associations, and policymakers.

Participants believed successful implementation would require collaboration across educational institutions, professional societies, healthcare organisations, and the Ministry of Health.

“Universities should introduce digital health earlier, professional associations should provide CPD, and the Ministry should recognise these competencies during licensure.”

(Occupational Therapist, 17 years)

Participants expressed generally favourable attitudes toward digital transformation and considerable willingness to engage with digital technologies when appropriate training and organisational support were available. The qualitative findings complemented the survey results by explaining why digital health, artificial intelligence, telerehabilitation, and virtual rehabilitation emerged as the highest-priority CPD topics across all rehabilitation professions. Table 4 below represent the joint Display Integrating Quantitative and Qualitative Findings.

## 4. Discussion

To our knowledge, this study is the first to examine digital health and AI-related continuing professional development needs across multiple rehabilitation professions in Jordan using a convergent mixed-methods approach. The findings demonstrated a substantial gap between rehabilitation professionals’ recognition of the importance of digital transformation and their preparedness to implement emerging technologies in clinical practice. Although participants reported overwhelmingly positive attitudes toward digital health and AI, relatively few had received formal education in these areas, and only one-third had previously attended digital health-related CPD. Collectively, the quantitative and qualitative findings suggest that the Jordanian rehabilitation workforce is highly motivated to embrace digital innovation but lacks structured educational opportunities, institutional support, and national competency frameworks necessary for successful implementation.

One of the most important findings was the exceptionally high demand for CPD related to AI, telerehabilitation, virtual reality, and clinical decision-support systems. Artificial intelligence received the highest priority score among all proposed educational topics, followed closely by telerehabilitation and virtual rehabilitation technologies. These findings are unsurprising considering the rapid digital transformation currently occurring across healthcare systems worldwide. Recent advances in AI, machine learning, wearable technologies, and remote monitoring have fundamentally altered rehabilitation practice by enabling personalized treatment planning, objective movement analysis, remote patient management, and data-driven clinical decision-making [1,7]. Consequently, rehabilitation professionals increasingly require competencies that extend beyond traditional clinical skills to include digital literacy, technology evaluation, data interpretation, and ethical application of AI-supported clinical tools.

The present findings are consistent with emerging international evidence demonstrating growing educational demand for digital health competencies among healthcare professionals. Several studies have reported that clinicians generally recognise the potential benefits of AI for improving healthcare quality, efficiency, and patient outcomes, yet simultaneously report insufficient knowledge, limited confidence, and inadequate formal education to support implementation in routine clinical practice. Similar findings have been reported among physicians, nurses, physiotherapists, and allied health professionals across Europe, North America, and Asia, where digital health has become an increasingly important component of continuing professional education. However, compared with professionals working in high-income countries, rehabilitation professionals in many low- and middle-income countries continue to experience fewer educational opportunities, more limited technological infrastructure, and slower implementation of digital innovations, further widening existing workforce competency gaps [1].

Recent applied research illustrates that technical performance alone does not guarantee successful clinical implementation. Machine-learning approaches have demonstrated promising performance in clinical decision-support applications, including adverse drug-event identification and hybrid systems combining machine-learning algorithms with expert rules [24,25]. However, translation of such technologies into clinical practice requires professionals who can interpret algorithmic outputs, recognise uncertainty and limitations, maintain human oversight, and integrate technology safely into clinical workflows. Recent policy-oriented work has similarly highlighted the importance of infrastructure, workforce training, organisational readiness, and information systems [26]. These observations reinforce the present findings that CPD programmes should extend beyond technology awareness to include critical appraisal, responsible AI, data governance, cybersecurity, clinical safety, and implementation readiness.

The qualitative findings provided valuable contextual explanations for these quantitative results. Participants consistently described digital transformation as inevitable and expressed strong enthusiasm for incorporating AI into rehabilitation practice. Nevertheless, they repeatedly emphasised that awareness of digital technologies should not be confused with competence. Many participants reported becoming familiar with AI through conferences, webinars, scientific publications, or social media rather than through structured educational programmes. This distinction between awareness and practical competence is particularly important because superficial familiarity with emerging technologies does not necessarily translate into safe or effective clinical implementation. Participants therefore advocated for competency-based educational programmes that prioritise practical application rather than theoretical knowledge alone.

The discrepancy between participants’ positive attitudes toward AI and their relatively low self-reported competence may be interpreted using the Technology Acceptance Model (TAM), which proposes that technology adoption is influenced primarily by perceived usefulness and perceived ease of use [27]. Participants in the present study appeared to perceive considerable potential value in AI-assisted rehabilitation, particularly regarding clinical decision support, documentation, patient monitoring, and service accessibility. However, their limited confidence and lack of previous training suggest relatively low perceived ease of use, thereby reducing readiness for implementation despite generally favourable attitudes. This interpretation was reinforced by the qualitative interviews, in which participants consistently described uncertainty regarding how AI technologies should be incorporated into everyday clinical decision-making despite expressing optimism regarding their future potential.

Another notable finding was the limited proportion of participants who had received formal education in AI or digital health despite mandatory CPD requirements within Jordan. Less than one-fifth of respondents reported previous formal AI education, while only approximately one-third had participated in digital health-related CPD activities. These findings suggest that current CPD provision has not evolved at the same pace as technological innovation. Madi and Hamzeh [5] similarly reported that Jordanian physiotherapists primarily participated in traditional educational activities, including workshops, journal reading, and workplace lectures, with relatively limited opportunities for specialised digital health education. The present study extends these observations beyond physiotherapy by demonstrating that similar educational gaps exist across multiple rehabilitation professions, indicating that the issue is systemic rather than profession-specific.

The interviews further highlighted that existing CPD programmes continue to focus predominantly on conventional clinical assessment and treatment techniques while providing comparatively little exposure to digital rehabilitation technologies. Participants repeatedly described the need for practical, simulation-based learning experiences involving telehealth platforms, wearable technologies, virtual reality systems, and AI-supported clinical applications. These preferences align with current evidence from health professions education demonstrating that experiential learning approaches, including simulation, case-based learning, and immersive technologies, improve self-reported digital health competency development more effectively than traditional didactic teaching alone. Consequently, future CPD initiatives should move beyond knowledge transmission toward competency-based educational models that allow rehabilitation professionals to apply emerging technologies within clinically relevant scenarios.

The present study also identified substantial organisational and personal barriers that limited participation in digital health-related CPD. Lack of time emerged as the most frequently reported barrier, followed by financial cost, limited availability of AI-focused educational programmes, inadequate employer support, and travel constraints. These findings closely mirror previous research conducted among Jordanian physiotherapists, in which time limitations, financial costs, and accessibility issues were identified as major obstacles to continuing professional development [5]. Similar barriers have also been consistently reported among healthcare professionals internationally, suggesting that workforce development is influenced not only by individual motivation but also by organisational capacity and healthcare system priorities.

The qualitative findings provided additional insight into these barriers by illustrating how heavy clinical workloads, staffing shortages, and competing service demands reduced professionals’ ability to engage in CPD, even when educational opportunities were available. Participants frequently described completing full clinical workloads before attempting to participate in evening or weekend educational activities, resulting in physical fatigue and reduced motivation for further learning. This finding highlights an important distinction between willingness to participate and the practical ability to do so. Simply increasing the number of available CPD programmes may therefore be insufficient unless healthcare organisations simultaneously address structural barriers such as protected learning time, workload management, and financial support.

Institutional support emerged as another important determinant of self-reported confidence. Participants described organisational expectations to adopt digital technologies while simultaneously reporting limited access to equipment, inadequate technical infrastructure, insufficient internet connectivity, and a lack of managerial support for professional development. These findings are particularly relevant within the context of Jordan’s national digital transformation strategy, which identifies workforce preparedness as one of the principal challenges to successful digital health implementation [8]. The findings therefore suggest that investment in digital infrastructure alone is unlikely to produce meaningful transformation unless accompanied by sustained investment in workforce education and organisational readiness.

An important finding of the quantitative analysis was that previous participation in digital health training represented the strongest independent predictor of self-reported confidence. Participants who had previously attended digital health-related CPD reported significantly higher self-reported digital health competency scores than those without previous training. This relationship remained statistically significant after adjustment for years of professional experience and employer support, indicating that structured educational exposure contributes independently to self-reported digital health competency development. These findings are encouraging because they suggest that digital competencies are modifiable rather than fixed professional characteristics. Consequently, appropriately designed CPD programmes may contribute to workforce readiness, although their effectiveness requires prospective evaluation for digital rehabilitation.

Years of professional experience also emerged as a significant predictor of self-reported confidence, although the relationship appeared more complex than initially anticipated. While experienced clinicians demonstrated greater confidence in clinical reasoning and patient management, qualitative interviews suggested that younger professionals were often more familiar with digital technologies acquired during undergraduate education or through self-directed learning. Conversely, more experienced practitioners frequently described greater confidence in evaluating the clinical relevance and limitations of new technologies. These complementary strengths suggest that future CPD programmes should encourage intergenerational learning, whereby digitally experienced younger clinicians and clinically experienced senior practitioners contribute different forms of expertise to collaborative professional development initiatives.

The finding that physiotherapists demonstrated significantly greater confidence in digital technologies than other rehabilitation professions warrants careful interpretation. Physiotherapy has historically been one of the earliest rehabilitation professions to adopt digital technologies, particularly in telerehabilitation, virtual reality, motion analysis, wearable sensors, and remote exercise monitoring. Consequently, physiotherapists may have had greater opportunities for exposure to digital innovation through both undergraduate education and clinical practice. However, this finding should not be interpreted as evidence that other rehabilitation professions require less investment in self-reported digital health competency development. On the contrary, occupational therapists, speech-language therapists, and prosthetists/orthotists increasingly utilise telepractice, assistive technologies, digital assessment tools, and AI-assisted interventions within contemporary rehabilitation services. National CPD strategies should therefore ensure equitable access to profession-specific digital education while simultaneously promoting interdisciplinary learning that reflects the collaborative nature of rehabilitation practice.

One particularly noteworthy finding from the qualitative interviews was participants’ consistent preference for practical, experiential learning over traditional lecture-based education. Rather than requesting additional theoretical courses, participants repeatedly advocated for simulation-based workshops, supervised clinical demonstrations, virtual reality laboratories, case-based learning, and opportunities to practise using digital technologies within realistic clinical scenarios. These findings are consistent with adult learning theory, which emphasises that professionals learn most effectively when educational experiences are directly relevant to their clinical practice, encourage active participation, and facilitate immediate application of newly acquired knowledge. Experiential approaches are also increasingly recognised within health professions education as effective strategies for developing digital competencies because they improve confidence, reduce technology-related anxiety, and facilitate behavioural change.

An equally important qualitative finding concerned participants’ perceptions of artificial intelligence as a clinical support tool rather than a replacement for professional judgement. Although media narratives frequently portray AI as a disruptive technology capable of replacing healthcare professionals, participants consistently rejected this perspective. Instead, they viewed AI as an adjunct capable of enhancing clinical reasoning, automating routine administrative tasks, improving documentation, monitoring rehabilitation progress, and supporting evidence-informed decision-making while preserving clinician responsibility for final treatment decisions. This distinction has important educational implications because CPD programmes should emphasise the collaborative relationship between clinicians and AI rather than presenting technology as an autonomous decision-maker. Such an approach may reduce professional resistance while promoting safe and ethically responsible implementation of AI within rehabilitation services.

The qualitative concerns regarding privacy, transparency, algorithmic bias, and responsibility for AI-supported decisions indicate that digital health education should extend beyond technical familiarity. Safe implementation requires competencies in clinical governance, patient safety, data protection, cybersecurity, interoperability, and critical evaluation of digital technologies. In AI-supported rehabilitation, professionals should also understand the importance of validating technologies in the intended clinical context, maintaining human oversight of AI outputs, documenting AI-supported decisions where appropriate, recognising uncertainty and limitations, and maintaining clear professional accountability for clinical decisions. These competencies should therefore be incorporated into future digital health and AI-related CPD programmes alongside practical technology skills.

Ethical considerations also featured prominently throughout the interviews. Participants expressed concerns regarding patient privacy, algorithmic bias, transparency of AI-generated recommendations, accountability for clinical errors, and data security. These concerns are consistent with the growing international literature on trustworthy AI, which emphasises that successful implementation depends not only on technical performance but also on ethical governance, transparency, fairness, and public trust. Importantly, participants did not perceive these ethical challenges as barriers to implementation but rather as educational priorities requiring explicit attention within future CPD programmes. Accordingly, ethics, governance, data protection, and responsible AI should be considered core competencies within future digital health education for rehabilitation professionals rather than optional supplementary topics.

The integration of the quantitative and qualitative findings represents one of the principal strengths of this study. The survey identified AI, telerehabilitation, virtual rehabilitation, and clinical decision support systems as the highest-priority CPD topics, while the qualitative interviews explained why these priorities emerged. Participants consistently described a workforce that was highly motivated to engage with digital transformation but constrained by limited educational opportunities, insufficient organisational support, and uncertainty regarding practical implementation. This convergence between datasets strengthens confidence in the study findings and illustrates the value of mixed-methods approaches for workforce needs assessment. Rather than relying solely on numerical indicators of educational demand, the qualitative data provided important contextual explanations that can guide the design of future CPD programmes and policy interventions.

The findings have important implications for rehabilitation education in Jordan. Undergraduate rehabilitation curricula should progressively integrate digital health competencies rather than treating them as optional or postgraduate topics. Core competencies should include telehealth practice, AI literacy, digital ethics, wearable technologies, virtual and augmented reality, electronic health records, data interpretation, cybersecurity, and critical appraisal of digital health technologies. Embedding these competencies during undergraduate education may better prepare graduates for contemporary practice; however, the effectiveness of such curriculum changes requires prospective evaluation. Professional associations also have an important role in supporting workforce development. Given that CPD has become mandatory for professional licensure in Jordan, professional organisations should expand their educational portfolios to include structured programmes focused on digital rehabilitation. Educational offerings should prioritise competency-based learning through simulation, case-based discussions, supervised practical workshops, and interdisciplinary learning experiences rather than relying predominantly on traditional lecture-based formats. Developing nationally accredited CPD modules in digital health and AI could also support standardisation of competencies across rehabilitation professions while facilitating equitable access to high-quality training.

At the policy level, the findings support the objectives of Jordan’s national digital health strategy, which recognises workforce capacity as a critical determinant of successful digital transformation. Investment in digital infrastructure alone is unlikely to achieve meaningful improvements in rehabilitation services unless accompanied by sustained investment in workforce education, organisational readiness, and supportive implementation policies. Policymakers should therefore consider developing a national digital-health competency framework for rehabilitation professionals that defines minimum competency standards across different career stages and professional disciplines. Self-reported confidence could represent one component of assessment within such a framework, alongside objective competency assessment, practical performance, and workplace-based evaluation where feasible. Such a framework could guide undergraduate curriculum development, professional licensure requirements, CPD accreditation, and workforce planning while ensuring consistency across healthcare institutions.

The findings also have broader relevance for other low- and middle-income countries undergoing digital transformation of their healthcare systems. Although technological innovation has accelerated globally, workforce preparedness remains highly variable, particularly in settings where access to educational resources and digital infrastructure is limited. The educational priorities identified in this study, including AI, telehealth, virtual rehabilitation, wearable technologies, and digital clinical decision support, are unlikely to be unique to Jordan. Consequently, the present findings may provide a useful framework for designing digital health CPD programmes in comparable healthcare systems across the Middle East and other resource-constrained settings.

Proposed Implementation Roadmap.

Short-term implementation (0–6 months). Universities and professional associations should conduct a structured audit of existing digital-health education and CPD provision. Priority introductory modules should include AI literacy, responsible use of AI, digital privacy, cybersecurity, telehealth, and critical appraisal of digital-health technologies. The principal resources required would include curriculum-development expertise, digital-health specialists, online learning infrastructure, and protected educator time.

Medium-term implementation (6–12 months). Universities should begin embedding foundational digital-health competencies within undergraduate rehabilitation programmes. Professional associations should pilot competency-based CPD modules using case-based learning, simulation, supervised practical activities, and blended delivery. Healthcare employers should introduce protected learning time and identify digital-health champions within rehabilitation departments. Required resources would include simulation facilities or digital platforms, educator time, technical support, and CPD accreditation.

Long-term implementation (12–24 months). The Ministry of Health, professional regulatory bodies, universities, professional associations, and healthcare organisations should consider collaborating on a national rehabilitation digital-health competency framework. The framework could define minimum competencies across career stages and guide curriculum development, CPD accreditation, professional development, and workforce planning.

Evaluation should include participation rates, completion rates, self-reported confidence, objective competency assessments where feasible, implementation fidelity, and longer-term indicators such as technology adoption, workflow integration, service accessibility, and patient outcomes. These latter outcomes should be evaluated prospectively rather than assumed from the present cross-sectional findings.

The findings provide evidence-informed priorities for the development of digital health education and CPD in Jordan; however, recommendations concerning national competency frameworks, curriculum reform, CPD accreditation, and professional regulation should be regarded as proposed policy directions rather than interventions whose effectiveness has been established by this cross-sectional study.

Several strengths should be acknowledged. First, this study is the first national mixed-methods investigation to examine digital health and AI-related CPD needs across multiple rehabilitation professions in Jordan, addressing an important gap in the regional literature. Second, the convergent mixed-methods design enabled comprehensive understanding by integrating quantitative and qualitative evidence, thereby enhancing the credibility and interpretability of the findings. Third, questionnaire development followed established psychometric procedures, including expert review, content validity assessment, pilot testing, and reliability evaluation, increasing confidence in the measurement of the study constructs. Finally, participants were recruited from multiple healthcare sectors and geographical regions, improving the diversity of perspectives represented within the study.

The study also has several limitations. First, the cross-sectional design provides a snapshot of CPD needs and therefore cannot establish causal relationships between participant characteristics and self-reported confidence. Second, the use of convenience and snowball sampling may have introduced selection bias, as professionals with greater interest in digital health may have been more likely to participate. Third, all quantitative measures relied on self-reported data, which may be influenced by recall bias or social desirability bias and may not fully reflect actual self-reported confidence. Fourth, although participants represented all major rehabilitation professions and healthcare sectors, the findings may not be fully generalisable to rehabilitation professionals practising outside Jordan or in substantially different healthcare systems. Finally, the rapid pace of technological development means that educational priorities related to AI and digital health are likely to evolve, necessitating periodic reassessment of workforce training needs. Because recruitment was conducted through multiple overlapping institutional and online channels, a unique denominator of eligible professionals who received the survey invitation could not be reliably established. Consequently, a conventional response rate was not calculated. This also prevented direct comparison of respondents and non-respondents and means that non-response and self-selection bias cannot be excluded. An additional limitation is that the quantitative questionnaire was administered only in English. Although English proficiency was required for questionnaire completion, this may have excluded or underrepresented rehabilitation professionals with limited English proficiency and may therefore have introduced language-related selection bias. Future studies should consider developing and validating an Arabic version of the questionnaire to improve accessibility and reduce potential language-related bias.

Future research should extend the present findings in several directions. Longitudinal studies are needed to evaluate changes in self-reported digital health competency over time as national digital health initiatives continue to develop. Intervention studies should examine the effectiveness of different educational approaches, including simulation-based learning, virtual reality training, blended learning, and AI-assisted educational platforms, in improving digital competencies among rehabilitation professionals. Future investigations should also evaluate the impact of enhanced self-reported digital health competency on clinician performance, patient outcomes, service accessibility, and healthcare efficiency. In addition, validation of the questionnaire developed in the present study across different countries and rehabilitation disciplines would facilitate international comparisons and contribute to the development of globally relevant digital-health competency frameworks that incorporate self-reported confidence alongside objective and performance-based assessment where feasible.

## 5. Conclusions

In conclusion, rehabilitation professionals in Jordan reported substantial demand for digital-health and AI-related continuing professional development, while also reporting limited previous training and significant organisational and time-related barriers. The findings suggest four complementary areas for action. First, universities should consider integrating foundational digital-health competencies, AI literacy, digital ethics, data protection, cybersecurity, and critical appraisal into undergraduate rehabilitation curricula. Second, professional associations and CPD providers should develop practical, competency-oriented postgraduate programmes using simulation, case-based learning, supervised practice, and blended delivery. Third, healthcare employers should consider providing protected learning time, technical support, appropriate digital infrastructure, and financial support for professional development. Fourth, policymakers and regulatory bodies should consider developing a national rehabilitation digital-health competency framework to guide education, CPD accreditation, and workforce development. These recommendations should be considered evidence-informed priorities rather than demonstrated causal interventions, as the present study was cross-sectional and relied on self-reported measures.

## Figures and Tables

**Table 1:** Characteristics of the quantitative study participants.

Characteristic	n	%
Profession		
Physiotherapist	212	45.9
Occupational therapist	96	20.8
Speech-language therapist	84	18.2
Prosthetist/orthotist	70	15.2
Age		
Mean age ± SD	34.8 ± 8.7	—
Gender		
Female	279	60.4
Male	183	39.6
Highest qualification		
Bachelor’s degree	241	52.2
Higher Diploma	41	8.9
Master’s degree	145	31.4
PhD	35	7.6
Years of professional experience		
1–5 years	126	27.3
6–10 years	137	29.7
11–15 years	98	21.2
>15 years	101	21.9
Healthcare sector		
Government	198	42.9
Private	176	38.1
Academic	46	10.0
NGO	42	9.1
Region		
North	77	16.7
Central	312	67.5
South	73	15.8
Previous digital-health CPD		
Yes	169	36.6
No	293	63.4
Formal AI education		
Yes	82	17.7
No	380	82.3

**Table 2:** Multivariable linear regression for self-reported digital health competency.

Predictor	B	SE	β	95% CI	*p*-Value	VIF
Previous digital-health CPD	0.42	0.06	0.41	0.30–0.54	<0.001	1.21
Years of professional experience	0.03	0.01	0.19	0.01–0.05	0.008	1.38
Employer support	0.27	0.07	0.24	0.13–0.41	<0.001	1.29
Age	0.01	0.01	0.07	−0.01–0.03	0.186	1.51
Female sex	0.04	0.06	0.03	−0.08–0.16	0.512	1.08
Private-sector employment	−0.08	0.06	−0.06	−0.20–0.04	0.192	1.17
Academic employment	0.13	0.08	0.08	−0.03–0.29	0.109	1.14

R^2^ = 0.41; adjusted R^2^ = 0.39; F = 45.72; *p* < 0.001.

**Table 3:** Summary of themes, subthemes, and representative quotations from the qualitative interviews.

Theme	Subtheme	Summary of Findings	Representative Quotation
Theme 1. Rehabilitation professionals recognised digital transformation as inevitable but felt inadequately prepared	1.1 Awareness exceeded competence	Participants demonstrated good awareness of digital health and AI concepts but reported limited practical skills and little formal education in their application.	*“We hear about artificial intelligence almost every week, but knowing about it and actually using it with patients are completely different things.”* *(Physiotherapist, 9 years)*
	1.2 Fear of being left behind	Many participants expressed concern that failure to develop digital competencies would negatively affect future professional practice and employability.	*“Healthcare is changing rapidly. If we continue practising exactly as we did ten years ago, we will eventually become outdated.”* *(Speech-Language Therapist, 15 years)*
Theme 2. Current CPD opportunities did not adequately address emerging technologies	2.1 Traditional CPD dominated available training	Existing CPD programmes mainly focused on conventional clinical skills, with limited attention given to digital health, AI, or virtual rehabilitation technologies.	*“Most courses still teach conventional assessment and treatment techniques. Very few discuss artificial intelligence or digital rehabilitation.”* *(Physiotherapist, 6 years)*
	2.2 Preference for experiential learning	Participants consistently preferred practical workshops, simulation, and hands-on demonstrations over lecture-based educational approaches.	*“Watching someone explain AI is not enough. We need to practise using these systems ourselves.” * *(Occupational Therapist, 8 years)*
Theme 3. Organisational and system-level barriers limited self-reported digital health competency development	3.1 Time and workload constraints	Heavy workloads and competing clinical responsibilities reduced opportunities to participate in CPD activities.	*“By the time my working day finishes, I simply don’t have the energy to attend another three-hour course.”* * (Speech-Language Therapist, 12 years)*
	3.2 Limited institutional support	Participants described insufficient organisational investment in protected learning time, infrastructure, technical support, and funding for CPD.	*“Management expects us to use digital technologies, yet there is no protected learning time or financial support for training.”* *(Prosthetist, 14 years)*
Theme 4. Artificial intelligence was viewed as a clinical support tool rather than a replacement for professional judgement	4.1 Improving efficiency and decision-making	Participants believed AI could improve clinical efficiency, automate documentation, monitor patient progress, and support evidence-based decision-making.	*“If AI could analyse patient progress and prepare reports automatically, I would have much more time to focus on treatment.” * *(Physiotherapist, 10 years)*
	4.2 Ethical and professional concerns	Despite recognising the benefits of AI, participants raised concerns regarding privacy, accountability, transparency, and algorithmic bias.	*“If an AI system recommends a treatment that harms a patient, who is responsible? These questions need to be answered before widespread implementation.”* * (Academic Participant)*
Theme 5. Participants advocated for a national digital rehabilitation CPD strategy	5.1 National competency standards	Participants supported establishing nationally recognised digital-health competency standards for rehabilitation professionals.	*“Every rehabilitation professional in Jordan should graduate with the same minimum digital competencies.” * *(Academic Participant)*
	5.2 Multi-sector collaboration	Successful implementation was perceived to require collaboration between universities, healthcare organisations, professional associations, and policymakers.	*“Universities should introduce digital health earlier, professional associations should provide CPD, and the Ministry should recognise these competencies during licensure.” * *(Occupational Therapist, 17 years)*

**Table 4:** Joint Display Integrating Quantitative and Qualitative Findings.

Quantitative Finding	Qualitative Finding	Integration/Meta-Inference
AI was the highest-priority CPD topic (4.71 ± 0.52).	Participants reported widespread awareness of AI but limited formal education and practical experience.	Convergence: high demand for AI training appears to reflect a gap between awareness and practical capability.
Telerehabilitation was highly prioritised (4.64 ± 0.61).	Participants requested practical training in telehealth platforms and remote patient management.	Convergence: quantitative demand was reinforced by specific qualitative requests for practical training.
Lack of time was reported by 84.4% of participants.	Participants described heavy clinical workloads, staffing shortages and fatigue after work.	Convergence: qualitative findings explain how workload limits participation despite favourable attitudes toward CPD.
Inadequate employer support was reported by 63.0%.	Participants described limited protected learning time, equipment, internet access and managerial support.	Convergence: organisational barriers were consistently evident across datasets.
Previous digital health CPD was associated with higher self-reported competency (β = 0.41, *p* < 0.001).	Participants described structured training as more useful than informal exposure through social media or webinars.	Convergence: formal educational exposure was viewed as important for developing practical confidence.
AI ethics received high educational priority.	Participants raised privacy, bias, transparency and accountability concerns.	Expansion: qualitative findings broaden the meaning of AI competency beyond technical skills to responsible and safe implementation.

## Data Availability

The data presented in this study are available on request from the corresponding author due to privacy/ethical restrictions.

## Supplementary Materials

The following supporting information can be downloaded at: https://www.mdpi.com/article/10.3390/healthcare14203403/s1: Table S1: Exploratory factor analysis of the questionnaire.

## Appendix A. Questionnaire

Continuing Professional Development Needs in Digital Health and Artificial Intelligence Among Rehabilitation Professionals in Jordan

Instructions

Thank you for participating in this study. The questionnaire aims to identify the continuing professional development (CPD) needs of rehabilitation professionals regarding digital health and artificial intelligence (AI).

There are no right or wrong answers. Please answer honestly based on your own experiences. The questionnaire will take approximately 15 to 20 min to complete.

Unless otherwise indicated, use the following scale:

1 = Strongly disagree

2 = Disagree

3 = Neutral

4 = Agree

5 = Strongly agree

Section 1. Demographic and Professional Information

- Age (years): ______
- Gender

1. □ Male
2. □ Female
3. □ Prefer not to say

Profession

1. □ Physiotherapist
2. □ Occupational Therapist
3. □ Speech-Language Therapist
4. □ Prosthetist
5. □ Orthotist
6. □ Other (please specify): __________

Highest qualification

- □ Bachelor’s
- □ Higher Diploma
- □ Master’s
- □ PhD
- □ Other

Years of professional experience

- □ 1–5
- □ 6–10
- □ 11–15
- □ >15

Primary workplace

1. □ Government hospital
2. □ Private hospital
3. □ Rehabilitation center
4. □ University
5. □ Private clinic
6. □ NGO
7. □ Other

Region

1. □ North
2. □ Central
3. □ South

Area of clinical practice (multiple answers permitted)

Have you previously attended CPD related to digital health?

1. □ Yes
2. □ No

Have you received any formal education about Artificial Intelligence?

- □ Yes
- □ No

Section 2. Current CPD Participation

How often have you participated in the following CPD activities during the past 12 months?

1 = Never

2 = Once

3 = Occasionally

4 = Frequently

5 = Very frequently

**Activity**	**1**	**2**	**3**	**4**	**5**
Workshops					
Conferences					
Online courses					
Webinars					
Journal clubs					
Reading scientific literature					
Simulation training					
Hospital in-service education					
Professional certification courses					

Section 3. Barriers to CPD

Please indicate your agreement.

**Item**	**1**	**2**	**3**	**4**	**5**
CPD activities are expensive.					
I have insufficient time.					
My workload prevents participation.					
Travel limits my attendance.					
My employer provides inadequate support.					
There are few digital health CPD opportunities.					
There are few AI-related CPD opportunities.					
Lack of internet/resources limits participation.					
Family responsibilities limit participation.					

Section 4. Digital Health Competencies

Rate your confidence.

1 = Not confident

5 = Highly confident

**Item**	**1**	**2**	**3**	**4**	**5**
Using electronic health records					
Conducting telehealth consultations					
Using rehabilitation mobile applications					
Using wearable sensors					
Interpreting digital outcome measures					
Protecting patient privacy in digital systems					
Evaluating digital rehabilitation technologies					
Selecting appropriate digital tools					
Troubleshooting common technology problems					

Section 5. Artificial Intelligence Competencies

**Item**	**1**	**2**	**3**	**4**	**5**
I understand the basic principles of AI.					
I understand AI terminology.					
I can identify AI applications in rehabilitation.					
I know the limitations of AI.					
I can critically evaluate AI-generated recommendations.					
I understand ethical issues related to AI.					
I understand patient data privacy when using AI.					
I feel confident using AI-assisted rehabilitation tools.					
I would use AI if appropriate training were available.					

Section 6. CPD Needs

How important is further training in the following topics?

1 = Not important

5 = Extremely important

**Topic**	**1**	**2**	**3**	**4**	**5**
Artificial Intelligence					
Machine learning					
Clinical decision support systems					
Virtual rehabilitation					
Augmented reality					
Telerehabilitation					
Remote patient monitoring					
Wearable technologies					
Motion analysis					
Digital outcome measures					
Data analytics					
Electronic health records					
Cybersecurity					
Ethics of AI					
Prompt engineering for healthcare					
Evidence-based digital rehabilitation					
Implementation of digital technologies					

Section 7. Preferred CPD Delivery

Which learning methods would you prefer? (Select all that apply.) □ Face-to-face workshops □ Online live courses □ Self-paced online modules □ Hybrid learning □ Virtual reality simulation □ Clinical mentoring □ Case-based learning □ Microlearning videos □ Mobile applications □ Professional conferences Preferred duration □ Half day □ One day □ Two days □ Weekly course □ Monthly sessions Preferred certification □ University certificate □ Professional society certificate □ Ministry-approved certificate □ International certification

## Appendix B. Semi-Structured Interview Schedule

Exploring Continuing Professional Development Needs Related to Digital Health and Artificial Intelligence Among Jordanian Rehabilitation Professionals

Interview Information

Participant ID: ____________

Profession: __________________________

Years of Experience: ____________

Sector: □ Government □ Private □ Academic □ NGO

Date: _______________

Interviewer: __________________

Interview Duration: ___________

### Appendix B.1. Opening Statement

Thank you for agreeing to participate in this interview. The purpose of this discussion is to better understand your experiences with continuing professional development (CPD), digital health, and artificial intelligence (AI) in rehabilitation practice. We are interested in your personal opinions and experiences. There are no right or wrong answers. Your responses will remain confidential and anonymized.

### Appendix B.2. Interview Schedule

**Domain**	**Main Question**	**Suggested Probes**	**Study Objective**
Professional Background	Could you briefly describe your professional role and current clinical practice?	Years of experience, specialty, workplace, patient population	Participant characteristics
CPD Experience	Tell me about your experience with continuing professional development during your career.	Types of CPD attended, motivation, frequency, most beneficial activities	Explore current CPD participation
Perceived CPD Needs	Which knowledge or skills do you feel you need most to improve your clinical practice?	Clinical skills, communication, leadership, research, digital health	Identify priority CPD needs
Digital Health Experience	How are digital technologies currently used in your workplace?	Electronic health records, telehealth, rehabilitation apps, wearable devices, virtual reality	Explore current digital health practice
Confidence in Digital Health	How confident do you feel using digital technologies in rehabilitation?	Situations where technology is used confidently or avoided	Explore perceived competency
Artificial Intelligence Awareness	What does artificial intelligence mean to you in rehabilitation practice?	Understanding of AI, examples, misconceptions, previous exposure	Assess AI awareness
AI Applications	In your opinion, how could AI improve rehabilitation services?	Clinical decision support, documentation, assessment, education	Explore perceptions of AI
Barriers	What factors prevent rehabilitation professionals from learning or using digital health and AI?	Time, cost, workload, infrastructure, internet, institutional support, confidence, language	Identify barriers
Facilitators	What would help rehabilitation professionals develop digital and AI competencies?	Funding, management support, protected learning time, mentoring, policy, university training	Identify facilitators
Preferred Learning Methods	If a national CPD programme on digital health and AI were developed, what should it look like?	Face-to-face, online, hybrid, simulation, workshops, duration, assessment, certification	Inform CPD programme design
Priority Training Topics	Which digital health or AI topics should be taught first? Why?	Telehealth, AI, VR, robotics, wearable sensors, digital assessment, ethics, cybersecurity	Identify curriculum priorities
Ethical Considerations	What concerns do you have regarding the use of AI in rehabilitation?	Patient privacy, bias, clinical responsibility, trust, transparency	Explore ethical issues
Future Workforce	How do you think digital health and AI will change rehabilitation over the next five to ten years?	Future roles, workforce preparedness, university education	Future workforce planning
Recommendations	What recommendations would you make to universities, professional associations, and policymakers regarding CPD?	Curriculum changes, mandatory CPD, national strategy, funding	Policy recommendations
Closing	Is there anything else you would like to add that we have not discussed?	Additional comments	Ensure completeness

### Appendix B.3. Interviewer Prompts

Could you tell me more about that?

Can you give me an example?

What happened next?

Why do you think that occurred?

Could you explain what you mean?

How did that affect your clinical practice?

Is that experience common among your colleagues?

Would you elaborate on that point?

### Appendix B.4. Field Notes

General impressions

Non-verbal observations

Contextual factors

Emerging ideas

Reflexive comments

Potential analytical memos

## References

[1] World Health Organization (2017). Rehabilitation 2030: A Call for Action, 6–7 February 2017, Executive Boardroom, WHO Headquarters: Meeting Report.

[2] Cieza A., Causey K., Kamenov K., Hanson S.W., Chatterji S., Vos T. (2020). Global estimates of the need for rehabilitation based on the Global Burden of Disease Study 2019: A systematic analysis for the Global Burden of Disease Study 2019. Lancet.

[3] World Health Assembly (2023). Strengthening Rehabilitation in Health Systems; Resolution WHA76.6, Agenda Item 13.4, 30 May 2023.

[4] World Health Organization (2021). Global Strategy on Digital Health 2020–2025.

[5] Madi M., Hamzeh H. (2022). Continuing professional development of Jordanian physiotherapists: A survey of activities, facilitators and barriers. Physiother. Pract. Res..

[6] Cottrell M.A., Galea O.A., O’Leary S.P., Hill A.J., Russell T.G. (2017). Real-time telerehabilitation for the treatment of musculoskeletal conditions is effective and comparable to standard practice: A systematic review and meta-analysis. Clin. Rehabil..

[7] Liu H., Xie Q. (2025). Applications of artificial intelligence in rehabilitation: Technological innovation and transformation of clinical practice. SLAS Technol..

[8] Melhem S.J., Nabhani-Gebara S., Bulatova N., Alabbadi I., Almomani H., Mousa R., Almousa A., Alrashdan Y., Nimri O., Kayyali R. (2025). Scaling holistic e-health solutions in cancer care using a qualitative realist framework. Front. Public Health.

[9] Aljabri N.Q., Bulkeley K., Cusick A. (2021). Telerehabilitation in the Middle East North Africa region: A structured review. Int. J. Telerehabil..

[10] Creswell J.W., Plano Clark V.L. (2018). Designing and Conducting Mixed Methods Research.

[11] Fetters M.D., Curry L.A., Creswell J.W. (2013). Achieving integration in mixed methods designs: Principles and practices. Health Serv. Res..

[12] O’Cathain A., Murphy E., Nicholl J. (2008). The quality of mixed methods studies in health services research. J. Health Serv. Res. Policy.

[13] von Elm E., Altman D.G., Egger M., Pocock S.J., Gøtzsche P.C., Vandenbroucke J.P. (2007). The Strengthening the Reporting of Observational Studies in Epidemiology (STROBE) statement: Guidelines for reporting observational studies. PLoS Med..

[14] Tong A., Sainsbury P., Craig J. (2007). Consolidated criteria for reporting qualitative research (COREQ): A 32-item checklist for interviews and focus groups. Int. J. Qual. Health Care.

[15] Patton M.Q. (2015). Qualitative Research & Evaluation Methods.

[16] Regmi K., Jones L. (2020). A systematic review of the factors–enablers and barriers–affecting e-learning in health sciences education. BMC Med. Educ..

[17] Guest G., Bunce A., Johnson L. (2006). How many interviews are enough? An experiment with data saturation and variability. Field Methods.

[18] Hennink M., Kaiser B.N., Weber M.B. (2019). What influences saturation? Estimating sample sizes in focus group research. Qual. Health Res..

[19] Cochran W.G. (1977). Sampling Techniques.

[20] Tabachnick B.G., Fidell L.S. (2019). Using Multivariate Statistics.

[21] Braun V., Clarke V. (2006). Using thematic analysis in psychology. Qual. Res. Psychol..

[22] Braun V., Clarke V. (2019). Reflecting on reflexive thematic analysis. Qual. Res. Sport Exerc. Health.

[23] Lincoln Y.S., Guba E.G. (1985). Naturalistic Inquiry.

[24] Toni E., Ayatollahi H., Abbaszadeh R., Fotuhi Siahpirani A. (2026). Identifying adverse drug events in children with congenital heart disease using machine learning techniques and multi-domain descriptors. Netw. Model. Anal. Health Inform. Bioinform..

[25] Toni E., Ayatollahi H., Abbaszadeh R., Fotuhi Siahpirani A. (2026). A clinical decision support system for predicting drug-related adverse effects in pediatric patients with congenital heart disease: Development and retrospective evaluation. J. Int. Med. Res..

[26] Toni E., Ayatollahi H. (2024). Addressing drug-related side effects in children with congenital heart disease: A policy brief. Glob. Pediatr. Health.

[27] Davis F.D. (1989). Perceived usefulness, perceived ease of use, and user acceptance of information technology. MIS Q..

