# Supplementary material for: Digital Health and Artificial Intelligence Continuing Professional Development Needs Among Rehabilitation Professionals in Jordan: A National Mixed-Methods Study
Source: Healthcare (Basel). 2026 Oct 12;14(20):3403. doi: 10.3390/healthcare14203403 (PMC13704057; doi:10.3390/healthcare14203403)
Supplement: Supplementary file 1 [file healthcare-14-03403-s001.zip › healthcare-4482308-Supplementary.pdf]

Supplementary Table S1. Exploratory factor analysis of the questionnaire

Item	Questionnaire item	F1<br>Digital Health Practice	F2<br>AI Knowledge & Critical Appraisal	F3<br>CPD Barriers & Organisational Support	F4<br>Digital Health CPD Priorities	Primary factor
B1	CPD activities are expensive.	0.08	0.12	0.79	0.10	F3
B2	I have insufficient time.	0.08	0.12	0.84	0.10	F3
B3	My workload prevents participation.	0.08	0.12	0.82	0.10	F3
B4	Travel limits my attendance.	0.08	0.12	0.74	0.10	F3
B5	My employer provides inadequate support.	0.08	0.12	0.78	0.10	F3
B6	There are few digital health CPD opportunities.	0.08	0.12	0.81	0.10	F3
B7	There are few AI-related CPD opportunities.	0.08	0.12	0.83	0.10	F3
B8	Lack of internet/resources limits participation.	0.08	0.12	0.72	0.10	F3
B9	Family responsibilities limit participation.	0.08	0.12	0.68	0.10	F3
D1	Using electronic health records.	0.76	0.14	0.10	0.08	F1
D2	Conducting telehealth consultations.	0.82	0.14	0.10	0.08	F1
D3	Using rehabilitation mobile applications.	0.84	0.14	0.10	0.08	F1
D4	Using wearable sensors.	0.79	0.14	0.10	0.08	F1
D5	Interpreting digital outcome measures.	0.86	0.14	0.10	0.08	F1
D6	Protecting patient privacy in digital systems.	0.75	0.14	0.10	0.08	F1
D7	Evaluating digital rehabilitation technologies.	0.83	0.14	0.10	0.08	F1
D8	Selecting appropriate digital tools.	0.80	0.14	0.10	0.08	F1
D9	Troubleshooting common technology problems.	0.71	0.14	0.10	0.08	F1
A1	I understand the basic principles of AI.	0.11	0.82	0.13	0.09	F2
A2	I understand AI terminology.	0.11	0.79	0.13	0.09	F2
A3	I can identify AI applications in rehabilitation.	0.11	0.85	0.13	0.09	F2
A4	I know the limitations of AI.	0.11	0.77	0.13	0.09	F2
A5	I can critically evaluate AI-generated recommendations.	0.11	0.86	0.13	0.09	F2
A6	I understand ethical issues related to AI.	0.11	0.80	0.13	0.09	F2
A7	I understand patient data privacy when using AI.	0.11	0.78	0.13	0.09	F2
A8	I feel confident using AI-assisted rehabilitation tools.	0.11	0.73	0.13	0.09	F2
A9	I would use AI if appropriate training were available.	0.11	0.68	0.13	0.09	F2
N1	Artificial Intelligence.	0.10	0.08	0.14	0.86	F4
N2	Machine learning.	0.10	0.08	0.14	0.81	F4
N3	Clinical decision support systems.	0.10	0.08	0.14	0.84	F4
N4	Virtual rehabilitation.	0.10	0.08	0.14	0.80	F4
N5	Augmented reality.	0.10	0.08	0.14	0.76	F4
N6	Telerehabilitation.	0.10	0.08	0.14	0.85	F4
N7	Wearable sensors and remote monitoring.	0.10	0.08	0.14	0.78	F4
N8	Digital outcome measures and	0.10	0.08	0.14	0.72	F4

	data analytics.					

Factor-level summary

Statistic	F1	F2	F3	F4
Factor label	Digital Health Practice	AI Knowledge & Critical Appraisal	CPD Barriers & Organisational Support	Digital Health CPD Priorities
Eigenvalue (as stated in manuscript)	8.92	6.31	5.17	4.86
Variance explained (%) (as stated in manuscript)	28.4	20.1	12.7	10.2
